# Aberrant PTPRO methylation in tumor tissues as a potential biomarker that predicts clinical outcomes in breast cancer patients

**DOI:** 10.1186/1471-2156-15-67

**Published:** 2014-06-11

**Authors:** Shao-ying Li, Rong Li, Yu-li Chen, Li-kuang Xiong, Hui-lin Wang, Lei Rong, Rong-cheng Luo

**Affiliations:** 1 Department of Breast Surgery, Bao’an Maternal and Child Health Hospital, Shenzhen, People’s Republic of China; 2 TCM-Integrated Cancer Center of Southern Medical University, 510515 Guangzhou, People’s Republic of China; 3 Department of Women’s Health, Bao’an Maternal and Child Health Hospital, Shenzhen, People’s Republic of China; 4 Central Lab, Bao’an Maternal and Child Health Hospital, Shenzhen, People’s Republic of China; 5 Department of Breast Surgery, ShenZhen Maternal and Child Health Hospital, Shenzhen, People’s Republic of China

**Keywords:** Protein tyrosine phosphatase receptor-type O (PTPRO), Methylation, Breast cancer, Clinical outcome, Biomarker

## Abstract

**Background:**

Aberrant hypermethylation of gene promoter regions is a primary mechanism by which tumor suppressor genes become inactivated in breast cancer. Epigenetic inactivation of the protein tyrosine phosphatase receptor-type O gene (*PTPRO*) has been described in several types of cancer.

**Results:**

We screened primary breast cancer tissues for *PTPRO* promoter hypermethylation and assessed potential associations with pathological features and patient outcome. We also evaluated its potential as a breast cancer biomarker. *PTPRO* methylation was observed in 53 of 98 (54%) breast cancer tissues but not in adjacent normal tissue. Among matched peripheral blood samples from breast cancer patients, 33 of 98 (34%) exhibited methylated *PTPRO* in plasma. In contrast, no methylated *PTPRO* was observed in normal peripheral blood from 30 healthy individuals. *PTPRO* methylation was positively associated with lymph node involvement (*P =* 0.014), poorly differentiated histology (*P =* 0.037), depth of invasion (*P =* 0.004), and *HER2* amplification (*P =* 0.001). Multivariate analysis indicated that aberrant *PTPRO* methylation could serve as an independent predictor for overall survival hazard ratio (HR): 2.7; 95% CI: 1.1-6.2; *P =* 0.023), especially for patients with HER2-positive (hazard ratio (HR): 7.5; 95% CI: 1.8-31.3; *P =* 0.006), but not in ER + and PR + subpopulation. In addition, demethylation induced by 5-azacytidine led to gene reactivation in *PTPRO*-methylated and -silenced breast cancer cell lines.

**Conclusions:**

Here, we report that tumor *PTPRO* methylation is a strong prognostic factor in breast cancer. Methylation of *PTPRO* silences its expression and plays an important role in breast carcinogenesis. The data we present here may provide insight into the development of novel therapies for breast cancer treatment. Additionally, detection of *PTPRO* methylation in peripheral blood of breast cancer patients may provide a noninvasive means to diagnose and monitor the disease.

## Background

Breast cancer is one of the most common cancers among women worldwide, and its incidence, unfortunately, continues to rise. Breast tumor is a heterogeneous disease derived from different molecular subtypes and displaying varied clinical behavior [1]. Considerable efforts have been made to improve survival via early diagnosis and treatment with targeted therapies [2]. However, the limited success of current therapeutic modalities has led to calls for new prognostic tools and for the development of additional targeted therapies [3].

Promoter hypermethylation is a type of epigenetic alteration associated with gene silencing. In cancer, many tumor suppressor genes are inactivated in this way. Hypermethylation of key tumor suppressors is a key contributor to breast tumorigenesis and acts in concert with genetic alterations to drive disease progression [4]. Epigenetic modifications of tumor DNA may have prognostic significance for breast cancer patients and provide targets for treatment because they are potentially reversible. Epigenetic changes may also serve as markers for early detection of the disease. As an example, screening for *RASSF1A* hypermethylation in serum has been proposed as a form of surveillance to detect early stage breast cancer [5].

In recent years, there has been considerable interest in better understanding the role of tyrosine phosphorylation in cancer [6-11], especially since this post-translational modification helps regulate diverse cellular processes, including proliferation, differentiation, metabolism, cell-to-cell communication, transcription, and survival [12]. Phosphorylation is a dynamic process that is positively regulated by protein tyrosine kinases (PTKs) and negatively regulated by protein tyrosine phosphatases (PTPs). More than 80% of oncogenes encode PTKs [13]; in contrast, many PTPs have been described to function as tumor suppressors [14]. For example, the tyrosine phosphatase PTPN2 activates TP53 and induces apoptosis in human tumor cells [15]. Another phosphatase, PTP1B, negatively regulates insulin signaling via dephosphorylation of insulin receptor kinase [16]. Computational analysis of the human genome identified 38 classical *PTP* genes, 19 of which mapped to regions frequently deleted in human cancers. Thirty of these protein phosphatases have been implicated in tumorigenesis [17], further demonstrating their potential roles as tumor suppressors.

Protein tyrosine phosphatase receptor-type O (*PTPRO*) is classified as a receptor-type PTP of the R3 subtype [18] and exhibits characteristics of a tumor suppressor in multiple cancers [19]. Several PTPRO variants have been described due to use of distinct transcriptional start sites and to alternative splicing; while many lymphoid-derived cells express a truncated PTPRO isoform, most epithelial tissues, including the breast, express the full-length form [19]. Previous studies have reported methylation-mediated down-regulation of PTPRO expression in breast cancer and other tumor types, such as rat hepatocellular carcinoma, human chronic lymphocytic leukemia, human lung cancer, esophageal carcinoma [6-11,20]. Hypermethylation of *PTPRO* occurs frequently in esophageal carcinoma and may be a potential biomarker of the disease [20]. A recent study also revealed that acute lymphoblastic leukemia patients with *PTPRO* methylation showed increased rates of relapse and chemoresistance [9].

More recently, a tumor suppressive role for PTPRO in breast cancer has emerged. Tumor-specific *PTPRO* promoter methylation was documented in primary human breast cancer cases [10]. The authors of this study also found that PTPRO expression was reduced upon treatment with estrogen but increased by treatment with the anti-estrogen Tamoxifen. Furthermore, ectopic expression of PTPRO in non-expressing MCF-7 cells sensitized them to the growth suppressive effects of Tamoxifen. PTPRO methylation has been further confirmed to be clinically relevant in breast cancer, particularly in *HER2*-amplified patients. Huang *et al*. showed that overall survival is significantly worse in HER2-positive patients with methylated PTPRO compared to tumors lacking methylation of this promoter region [21]. Another study found that low expression of PTPRO correlated with reduced survival for HER2-positive breast cancer patients [11]. It is possible that the pronounced impact of PTPRO specifically in HER2-positive disease could be due to the fact that HER2 itself is a direct substrate of PTPRO phosphatase activity [11]; specifically, loss of PTPRO was shown to increase HER2 phosphorylation and HER2-induced proliferation and transformation of breast cancer cell lines. Taken together, these data support a role for PTPRO as a tumor suppressor in breast cancer and suggest that its methylation and expression may have prognostic significance in the disease.

In the current study we investigated the methylation status of *PTPRO* in primary human breast cancer from fresh frozen specimens with the aim of defining the frequency of this epigenetic aberration in the disease. We examined the methylation status of *PTPRO* in primary breast tumors and matched peripheral blood samples and determined if promoter methylation was associated with decreased gene expression in breast cancer cell lines. We also examined associations between *PTPRO* methylation and several clinicopathological parameters, including patient outcome.

## Methods

### Tumor samples

Between 2006 and 2009, we obtained 98 tumor samples and matched pre-operative peripheral blood samples from women undergoing surgery for primary invasive breast carcinoma at ShenZhen Maternal and Child Health Hospital, an affiliate of Southern Medical University in China. None of the patients had received any pre-operative treatment, including chemotherapy or radiotherapy. This is a well-characterized series of patients under the age of 74 years (median, 46 years). The median follow-up time of patients in the study was 60 months (range 43–70 months). All patients were treated uniformly at a single institution.

Pathologic characteristics, including histological grade, histological tumor type, tumor size, and lymph node involvement were routinely assessed; several patient characteristics, including age and family history of cancer and menopause, were also recorded. Survival data were maintained prospectively. At the end of the study period, 39 (40%) patients had died because of disease recurrence. In total, 98% of node-positive and 82% of node-negative patients received adjuvant systemic therapy consisting of either hormone therapy alone or hormone therapy plus chemotherapy.

Tumor samples were immediately frozen in liquid nitrogen and stored at −80°C until use. All tumors were confirmed histopathologically and their clinical features were classified based on the TNM system of the International Union Against Cancer [22]. Corresponding adjacent non-cancerous tissues were also obtained from surgical resections. Peripheral venous blood samples from breast tumor patients were collected in EDTA-containing tubes and immediately centrifuged at 2500 g for 15 min to prepare plasma. The plasma samples were stored at −80°C until further processing. Peripheral blood samples from an additional 30 healthy volunteers were used as normal controls. Estrogen receptor (ER), progesterone receptor (PgR), and human epidermal growth factor receptor 2 (HER2) immunohistochemistry was performed on TMA sections as previously described [23].

Approval for the use of human tissues and clinical information was obtained from the Committee for Ethical Review of Research involving Human Subjects at Southern Medical University. All patients provided written informed consent for sample collection prior to surgery.

### Cell culture and treatment

Human breast cancer cell lines MCF-7, MDA-MB-231, and Hs578t (provided by Dr. Qi T. Yan, Southern Medical University, Guangzhou, China), were maintained in DMEM supplemented with 5% fetal bovine serum and 1 mM nonessential amino acids in a 5% CO_2_ incubator. Normal human mammary epithelial cells (HMEC 48R; provided by Dr. Qi T. Yan, Southern Medical University, Guangzhou, China) were maintained in MEGM (Cambrex Corp., USA) as previously described [10]. To confirm that methylation of the *PTPRO* promoter in breast cancer cell lines was responsible for its suppression, MCF-7 and MDA-MB-231 cells were treated with 5-azacytidine (5-AzaC, Sigma Chemical Co., HK), a DNA-hypomethylating agent, according to the following conditions: 1 μmol/L for 72 h for MCF-7 cells, and 2.5 μmol/L for 96 h for MDA-MB-231 cells. The response of different cell lines to demethylating agents probably varies due to different drug sensitivities as well as different kinetics of association/dissociation of chromatin remodelers with specific genes. All cells used in this study were between passages 8 and 11.

### DNA extraction and bisulfite modification

Genomic DNA from primary tumors and plasma was extracted using a QIAamp DNA Mini Kit (Qiagen, Germany) and QIAamp DNA blood Mini Kit (Qiagen, Germany). Gene methylation status was evaluated using sodium bisulfite modification of DNA and subsequent methylation-specific PCR (MSP), essentially as previously described [24-26]. DNA (1–2 μg) from each sample was subjected to bisulfite modification using EpiTect 96 Bisulfite Kits according to the manufacturer’s instructions (Qiagen, Germany). Bisulfite-modified DNA was typically immediately used for PCR.

### Methylation-specific PCR analysis

Primer sequences for PCR amplification of methylated and unmethylated alleles of PTPRO were previously published [10] and are listed in Table 1. Primers were synthesized by Shenggong (Shenggong Biotech, Shanghai, China). Primers were designed to amplify 170 bp (methylated) or 201 bp (unmethylated) regions of the CpG island within the PTPRO promoter [8]. For each reaction, 3 μl of sodium bisulfite- converted DNA was added to a total volume of 50 μl of PCR mix (EpiTect MSP Kits, Qiagen, Germany) according to the manufacturer’s instructions. Briefly, samples were initially incubated at 95°C for 10 min. This was followed by 35 cycles of denaturation at 95°C for 15 s, annealing at 55°C for 30 s, and extension at 72°C for 30 s; finally, there was one round of extension at 72°C for 10 min. An additional 15 cycles of denaturing (30 s at 94°C), annealing (15 s at 50.4°C), and extension (30 s at 72°C) were required for blood samples. PCR products were analyzed by electrophoresis on 2% agarose gels. Primers for unmethylated PTPRO (Table 1) were used to confirm the presence of DNA in each sample following bisulfite modification. This control was run for each sample on the same day that MSP analysis was carried out for the PTPRO gene. Breast tumor samples previously identified as DNA hypermethylated were used as positive controls. For each PCR assay, experimental reactions were accompanied by a black reaction (no DNA), a negative control reaction (blood DNA), and a positive control reaction (breast cancer DNA).

**Table 1:** **PCR primer sequences for methylation analysis of *PTPRO***

**Primers**	**Sequences**	**Product size**
PTPRO-forward	5′-CTCCACCCAAATCACTCTTCGCAG-3′	268 bp
PTPRO-reverse	5′-ACCATTGTTGAGACGGCTATGAACG-3′	
18 s rRNA-forward	5′-TCAAGAACGAAAGTCGGAGG-3′	110 bp
18 s rRNA- reverse	5′-GGACATCTAAGGGCATCACA-3′	
MSP-methylated-forward	5′-CGTTTTTGGAGGATTTCGGGC-3′	170 bp
MSP-methylated- reverse	5′-AAAACACGACTACGCTAACG-3′	
MSP-unmethylated-forward	5′-ATGTTTTTGGAGGATTTTGGGT-3′	201 bp
MSP-unmethylated- reverse	5′-ATACCCCATCACTACACAAACA-3′	

### Bisulfite genomic sequencing

Bisulfite-converted DNA was used to PCR amplify the PTPRO CpG island from -208 bp to +236 bp with respect to the transcription start site as described earlier; ref. [7,8,19]. The PCR product was purified using a gel extraction kit (Qiagen, Germany). The purified PCR product was used for bisulfite sequencing and was cloned into the pDrive vector according to the instructions of the PCR cloning kit (Qiagen, Germany). Ten randomly selected clones were subjected to automated sequencing. Direct sequencing was performed using the Thermo Sequenase Radiolabeled terminator cycle sequencing kit (Qiagen, Germany) with the primer hGlepp1-BS-F3 (5′-TAGGGGGATTGGAAAGGTAG-3′) following the manufacturer’s protocol.

### RNA isolation and reverse transcription PCR analysis

Total RNA was isolated using the RNeasy Mini kit (Qiagen, Germany). Reverse transcription of deoxyribonuclease-treated RNA (1 μg) was carried out according to instructions provided with the QuantiTect Reverse Transcription kit (Qiagen, Germany). Semi-quantitative PCR for PTPRO expression was performed. 0.2 mM of each primer was added to a 25 μl PCR reaction mixture. Cycling conditions were as follows: denaturation at 94°C, annealing at 54.5°C (for PTPRO) or 65°C (for 18S rRNA), and extension at 72°C. For PTPRO, a total of 32 cycles were run, and for18S rRNA, 25 cycles were used. The PCR products were separated on 2% agarose, stained with ethidium bromide, and imaged under UV light using Bio-rad Quantity One software. 18S rRNA transcripts in each sample were also amplified as internal controls for normalization. Gene-specific primers used for amplification of PTPRO and 18S rRNA are listed in Table 1.

### Statistical analysis

The χ2 test was used to determine associations between methylation of *PTPRO* and various phenotypic or molecular features of breast cancer. Fisher’s exact test was used when individual cell numbers were less than 5. All P values were derived from two-tailed statistical tests and significance was assumed at P < 0.05. Kaplan–Meier analysis was used to assess cumulative survival probabilities, and differences were evaluated using the log-rank test. Multinominal logistic regression analyses were used to assess the hazard model for the survival of breast cancer patients. All analyses were performed using the SPSS 19.0 (Chicago, IL, US) statistical software package.

## Results

### Frequent methylation of PTPRO in primary tumors and peripheral blood samples

**Figure 1:**
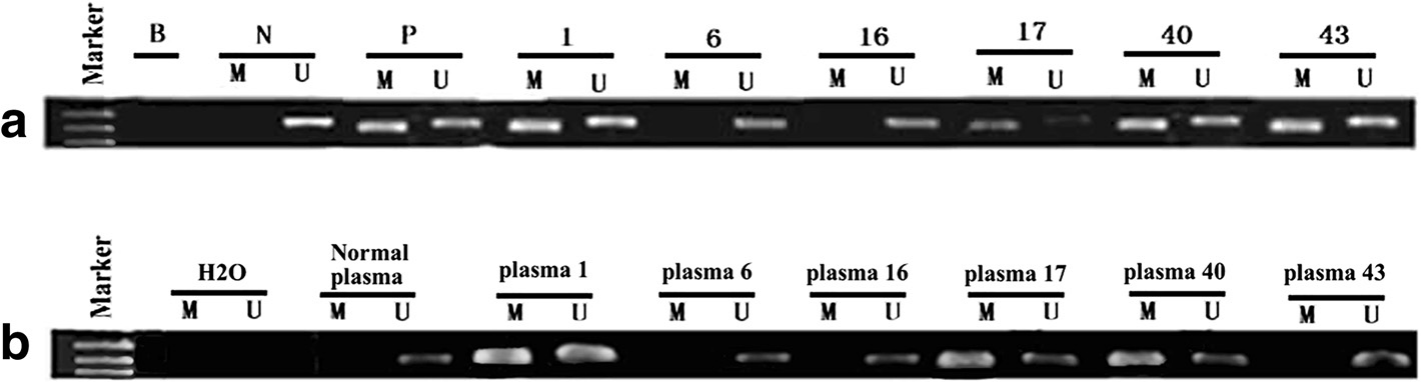
**Representative MSP results for methylation of the *PTPRO *gene. (a)** primary breast tumors; **(b)** matched peripheral blood samples. Numbers indicate the sample number. B, blank (no DNA); N, negative control; P, positive control; M, methylated; U, unmethylated.

Methylation of the *PTPRO* promoter has been demonstrated in different tumor types, including breast cancer [7,8,10]. These observations, along with the growth-suppressive properties not only of PTPRO [7,8] but of PTPs in general [27], prompted us to further investigate *PTPRO* methylation status in a large series of human breast tumors. Genomic DNA isolated from tumor tissue, surrounding normal tissue, and matched peripheral blood samples (n = 98) was subjected to MSP analysis. Among the 98 primary breast tumor specimens investigated, 53 (54%) showed hypermethylation of *PTPRO*. Methylation of this gene was not observed in any adjacent normal tissues. 33 of 98 (34%) patients exhibited detectable levels of methylated *PTPRO* in matched plasma. No methylated PTPRO was observed in normal peripheral blood samples from 30 healthy individuals. The χ2 test revealed that *PTPRO* methylation in plasma was significantly correlated to that in tumor tissue (*r* = 0.435; *P* < 0.0001, Table 2). Representative MSP results from primary tumors are shown in Figure 1a, b.

**Table 2:** Association of methylation of PTPRO gene between tumor tissues and plasma

**Tumor tissues**	**Plasma**		
	**M**	**U**	**Total**
M	29	24	53
U	4	41	45
Total	33	65	98

M: Methylated; U: Unmethylated.

### Clinicopathological significance of PTPRO methylation in breast tumors and peripheral blood samples

Associations between *PTPRO* methylation and clinicopathological and molecular features of breast tumors in this study are shown in Table 3. The strongest correlation was between *PTPRO* methylation and *HER2* amplification (*P =* 0.001). *PTPRO* methylation was also significantly more frequent in node positive (*P =* 0.014), poorly differentiated (*P =* 0.037), and stage III (*P =* 0.004) tumors. The highest frequency of *PTPRO* methylation (84%) was seen in late stage tumors. Trends were also observed for more frequent *PTPRO* methylation in ER- and PR-negative tumors but no associations were apparent with patient age, tumor size, histological tumor type, or *TP53* mutation. Methylation of *PTPRO* in plasma was more frequent in those with *HER2* amplification (*P =* 0.018). For all other clinical and pathological parameters, there was no statistically significant correlation associated with methylation of *PTPRO* in plasma (Table 3).

**Table 3:** **Associations between *PTPRO *** **methylation and clinicopathological features of breast cancer**

**Characteristics**	**No.**	***PTPRO *** **methylation**					
		**Tumor tissue (%)**	χ^**2**^	***P *** **value**	**plasma (%)**	**χ**^**2**^	***P *** **value**
Total	98	53 (54)			33 (34)		
Age							
< 45 years	45	22 (49)			16 (36)		
≥ 45 years	53	31 (59)	0.903	0.417	17 (32)	0.132	0.716
Nodal involvement							
Negative	61	27 (44)			18 (30)		
Positive	37	26 (70)	6.273	0.014	15 (40)	0.935	0.334
Stage							
I/II	79	37 (47)			24 (30)		
III	19	16 (84)	8.616	0.004	9 (47)	1.979	0.159
Histological type							
Non-ductal	23	12 (52)			7 (30)		
Ductal	75	41 (55)	1.000	0.510	26 (35)	0.141	0.707
Tumour size							
≤20 mm	47	26 (55)			12 (26)		
>20 mm	51	27 (53)	0.056	0.842	21 (40)	2.234	0.135
Histological grade							
Well/mod diff.	60	27 (45)			18 (30)		
Poorly diff.	38	26 (68)	5.139	0.037	15 (40)	0.935	0.334
ER status							
Negative	24	16 (67)			10 (42)		
Positive	74	37 (50)	2.027	0.167	23 (31)	0.909	0.340
PR status							
Negative	29	20 (69)			10 (35)		
Positive	69	33 (48)	3.674	0.076	23 (33)	0.012	0.912
*HER2* status							
Normal	51	19 (37)			12 (24)		
Amplified	47	34 (72)	12.124	0.001	21 (46)	5.570	0.018
*TP53* status							
Normal	59	30 (51)			17 (29)		
Mutant	39	23 (59)	0.624	0.535	16 (41)	1.568	0.211

### Prognostic significance of tumor tissue and plasma PTPRO methylation

**Figure 2:**
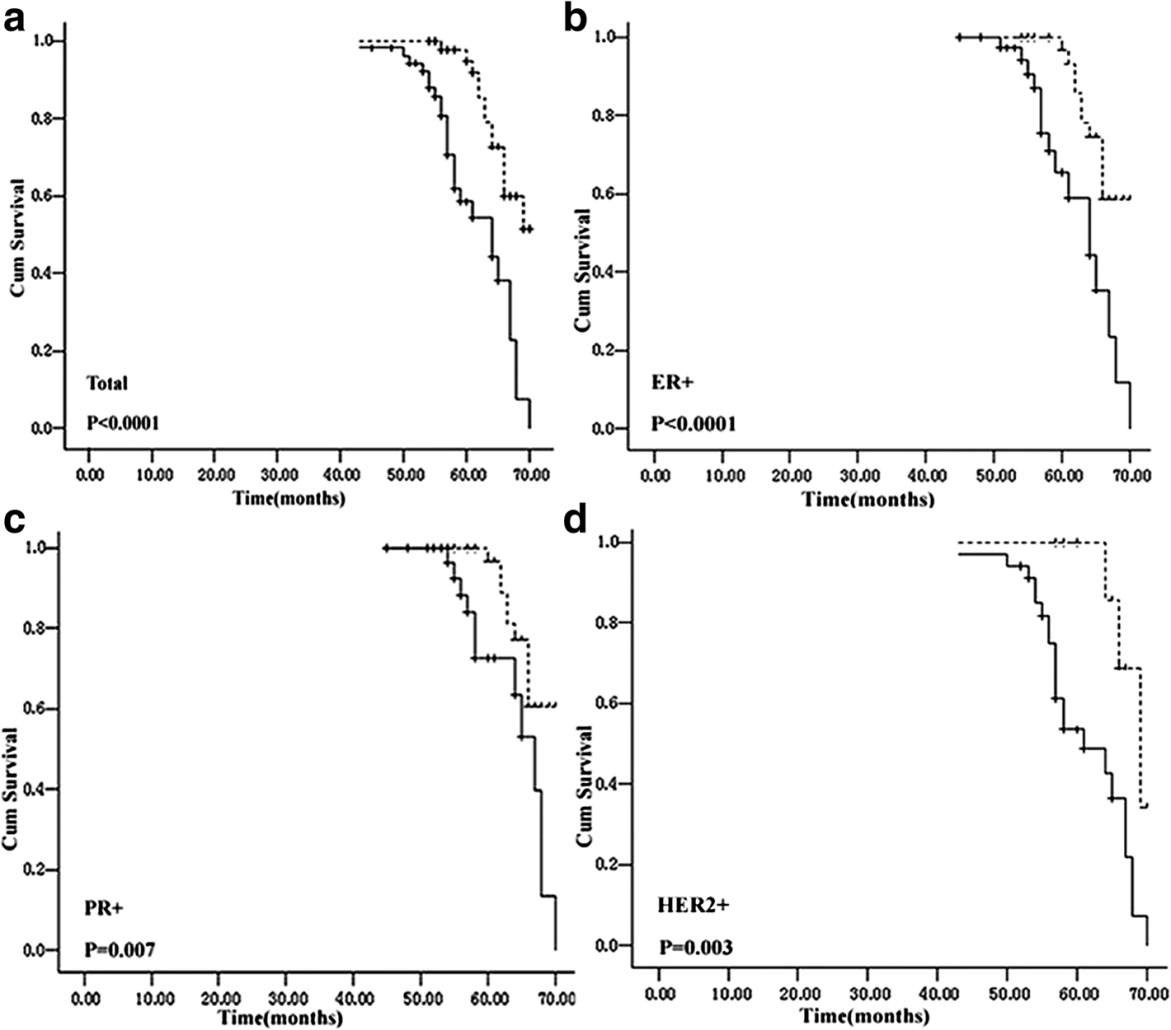
**Kaplan–Meier survival analysis for breast cancer patients with (solid line) *PTPRO *tumor methylation or without (dotted line). (a)** overall group; **(b)** ER+; **(c)** PR+; **(d)** HER2-amplified subgroup.

Univariate analysis examined clinicopathologic parameters including PTPRO methylation and their association with overall survival end points. The results showed that survival was significantly worse in patients with lymph node involvement (*P =* 0.0001), late stage tumors (*P =* 0.0001), poorly differentiated tumors (*P =* 0.033), larger tumors (*P =* 0.019), positive *HER2* amplification (*P =* 0.022), *TP53* mutation (*P =* 0.012) and PTPRO methylation (hazard ratio (HR): 3.8; 95% CI: 1.9-7.5; *P =* 0.0001; Table 4). We then stratified all patients into subpopulations according to ER, PR and HER2 status. In ER- positive, PR- positive and HER2-positive patients, the methylated PTPRO group show significantly worse overall survival compared to those of unmethylated PTPRO (*P* = 0.001, *P* = 0.012 and *P* = 0.010, respectively, Table 4). Kaplan-Meier curves for overall tumor group and the above subgroups according to PTPRO methylation are shown in Figure 2. As shown, tumor tissue *PTPRO* methylation was associated with significantly worse cancer-specific survival in the overall tumor group (log-rank test *P =* 0.0001; Figure 2a). Subgroup analysis revealed that *PTPRO* methylation also showed significant prognostic value within the ER + (*P =* 0.0001), PR + (*P =* 0.007), and *HER2*-amplified (*P =* 0.003) patient groups (Figure 2b, c, and d, respectively).

**Table 4:** Univariate and multivariate cox proportional hazard model for the survival of breast cancer patients

**Variable**	**Univariate analysis**			**Multivariate analysis**		
	**Hazard ratio**	**95% CI**	***P***	**Hazard ratio**	**95% CI**	***P***
Tumor size (large *vs* small)	2.4	1.2-4.9	0.019			
Lymph node status (pos. *vs* neg.)	4.9	2.4-9.8	0.0001	4.0	1.6 - 9.9	0.003
Histological grade (poor *vs* well)	2.1	1.1-4.0	0.033			
Stage (III *vs* I/II)	3.2	1.7-5.9	0.0001			
*HER2* status (amp. *vs* wildtype)	2.2	1.1-4.2	0.022			
*TP53 (*mutant *vs* wildtype)	2.3	1.2-4.3	0.012			
Tumor tissue *PTPRO* methylation (yes *vs* no)	3.8	1.9-7.5	0.0001	2.7	1.1- 6.2	0.023
ER + group tumor tissue PTPRO methylation (yes *vs* no)	3.9	1.7-8.7	0.001	2.8	1.0-8.4	0.060
PR + group tumor tissue PTPRO methylation (yes *vs* no)	3.1	1.2-7.4	0.012	3.2	0.8-11.9	0.091
HER2+ group tumor tissue PTPRO methylation (yes *vs* no)	5.0	1.8-16.8	0.010	7.5	1.8-31.3	0.006

To confirm the significance of this finding, we performed multivariate analysis, treating methylated-PTPRO as a factor with tumor size, lymph node metastasis, histological grade, stage, HER2 status and TP53 status for their impact on overall survival. After adjustment for these convariates, methylated-PTPRO was identified as an independent predictor for overall survival in all tumor group (hazard ratio (HR): 2.7; 95% CI: 1.1-6.2; *P =* 0.023) and HER2+ subpopulation (hazard ratio (HR): 7.5; 95% CI: 1.8-31.3; *P =* 0.006), but not in ER + and PR + subpopulation. Similarly, lymph node metastasis also had an independent association with overall survival in this patient series. We also analyzed the potential prognostic value of plasma *PTPRO* methylation but no significant data were obtained.

### PTPRO expression is inversely correlated with methylation status

**Figure 3:**
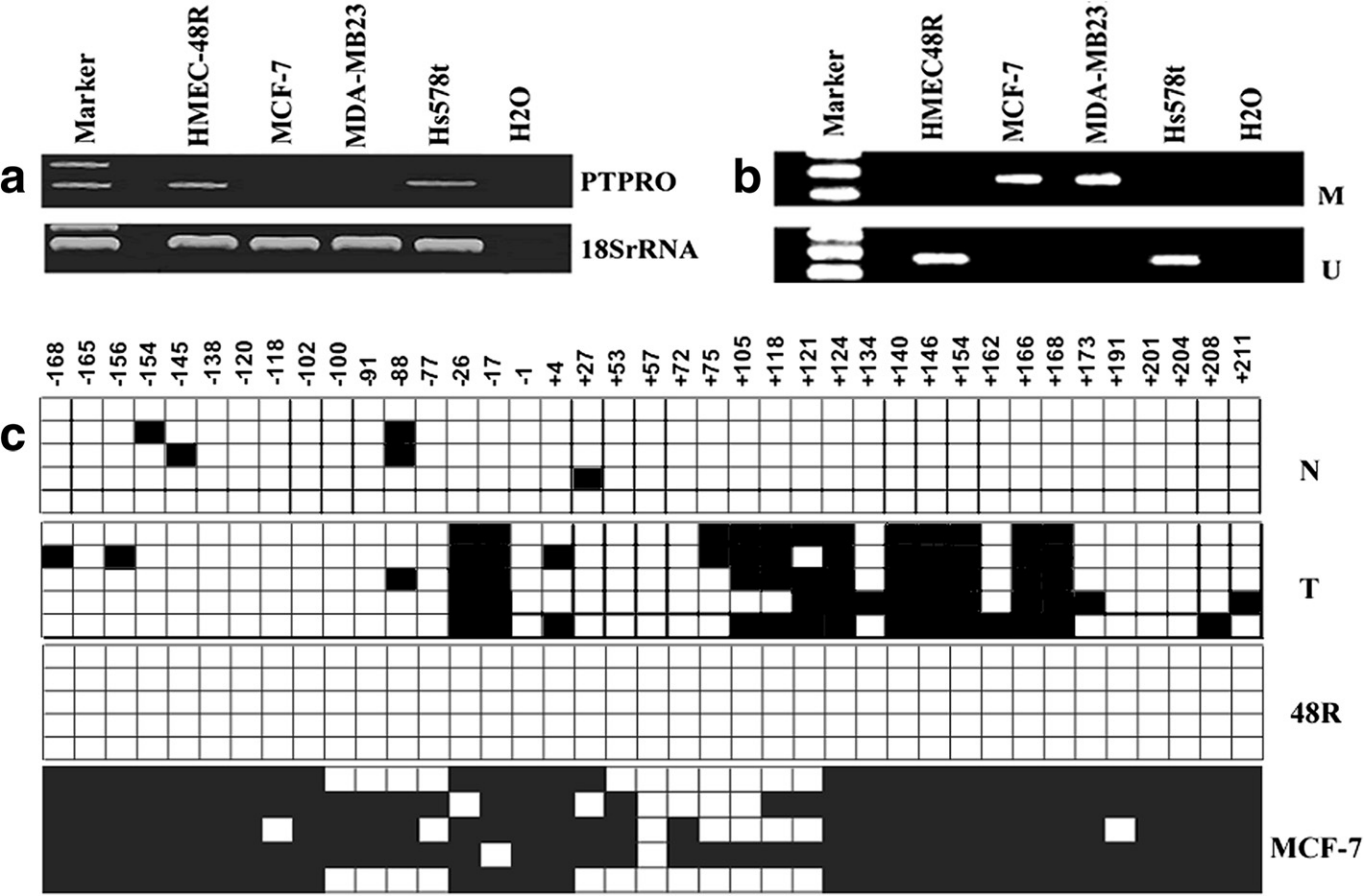
***PTPRO *is methylated in breast cancer cell lines but not in normal breast epithelial cells. (a)** Expression of PTPRO in normal human mammary epithelial cells (48R) and human breast cancer cell lines Hs578t, MCF-7, and MDA-MB-231. Total RNA isolated from cell lines was subjected to RT-PCR analysis using PTPRO-specific primers. 18S rRNA was used as an internal loading control. **(b)** MSP analysis of PTPRO methylation status in breast cancer cell lines. HMESC48R was used as a normal control. M, methylated; U, unmethylated. **(c)** PTPRO CpG island from randomly selected breast tumor tissue and its matched normal tissue; also shown are HMEC 48R and MCF-7 cells, all of which were subjected to BS genomic sequencing. Each solid square represents a methylated cytosine and an open square represents unmethylated cytosine in a CpG dinucleotide. Each row corresponds to a single clone. N, normal corresponding adjacent non-cancerous tissue; T, tumor tissue.

We next sought to determine the relationship between *PTPRO* methylation and gene expression in a panel of breast cancer cell lines (MCF-7, MDA-MB-231, and Hs578t) and in normal human mammary epithelial cells (HMEC, 48R). In normal mammary epithelial cells, PTPRO is expressed at appreciable levels and its promoter region is not methylated; in contrast, PTPRO expression was relatively low in two (MCF-7, MDA-MB-231) of the three breast cancer cell lines examined and its promoter was methylated (Figure 3a, b).

We performed bisulfite genomic sequencing from ten pairs of breast tumor tissue and matched normal tissue, one representative HMEC (48R), and one breast cancer cell line (MCF-7) to determine if the CpG Island located in the promoter of *PTPRO* was differentially methylated. Complete bisulfite conversion was confirmed by the presence of substituted thymine for all cytosine residues at non-CpG sites. We detected hypermethylation of CpGs in both *PTPRO*-silenced tumors and in MCF-7 cells. In contrast, the *PTPRO*-expressing cell line HMEC 48R and matched normal breast tissue exhibited low levels or no methylation of the *PTPRO* promoter—strongly supporting the MSP results (Figure 3c).

**Figure 4:**
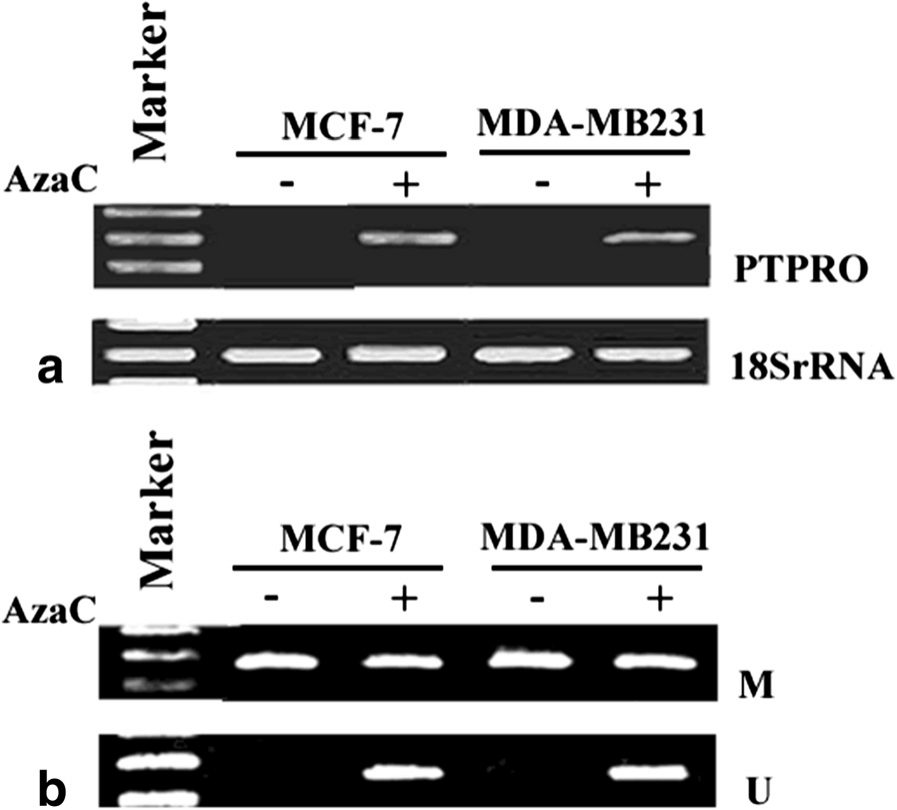
**Re-expression of PTPRO following treatment with 5-AzaC. (a)** Breast cancer cell lines MCF-7 and MDA-MB-231 were treated with 1 μM 5-AzaC for 72 h and 2.5 μM 5-AzaC for 96 h, respectively. Total RNA from cells was subjected to RT-PCR to amplify PTPRO mRNA. 18S rRNA was used for normalization; **(b)** MSP analysis of PTPRO methylation status in breast cancer cell lines with or without 5-AzaC treatment. M, methylated; U, unmethylated.

To confirm that hypermethylation of the *PTPRO* promoter was responsible for its suppression, both MCF-7 and MDA-MB-231 cells (hypermethylated PTPRO promoter; silenced mRNA expression) were treated with 5-AzaC at a final concentration of 1 μM for MCF-7 cells and 2.5 μM for MDA-MB-231 cells. Re-expression of PTPRO in both cell lines was observed after exposure to this demethylating agent for 72 h and 96 h, respectively (Figure 4a). Moreover, the MSP result showed that unmethylated PTPRO alleles increased after 5-AzaC treatment (Figure 4b). These data further support the notion that methylation of the *PTPRO* CpG island plays an important role in gene silencing.

## Discussion

Although protein tyrosine kinases have long been recognized as key players in oncogenesis, the role of protein tyrosine phosphatases in the initiation and progression of cancer is only now gaining increased attention [27-29]. In this study, the breast cancer series investigated here for DNA methylation is well characterized and conventional pathological indicators, including nodal involvement, histological grade, tumor size, and stage, all show the expected prognostic significance. *PTPRO* methylation was detected in two of three breast cancer cell lines and in 53 of 98 (54%) primary human breast cancer specimens; however, no *PTPRO* methylation was observed in adjacent normal tissue. This result is within the range (52% to 81%) reported in previous studies of human cancers [7-10,20,30]. The rather high frequency of methylation suggests that *PTPRO* is a common target for epigenetic silencing in breast tumors and that it may contribute to the development of this tumor type. As reported previously, demethylation of the *PTPRO* promoter resulted in gene re-expression [31]. These observations demonstrate growth-suppressor characteristics of PTPRO that are typical of a classical tumor suppressor gene.

Aberrant hypermethylation of tumor suppressor genes is an important epigenetic event in the development and progression of many human cancers and may serve as a biomarker for disease detection at early stages [32-34]. In this study, we detected *PTPRO* methylation in the plasma of 34% (33/98) of patients; this value was significantly correlated with *PTPRO* methylation detected in tumor tissue. Such a high correlation confirmed that peripheral blood samples could potentially be used to assist the detection and diagnosis of breast cancer. Moreover, this assay appears to be robust and highly specific; no methylated *PTPRO* was detected in plasma from breast cancer patients without primary tumor methylation or from normal healthy control peripheral blood samples. These findings are consistent with results published by Huang *et al*. [21] who also examined PTPRO methylation in peripheral blood samples from breast cancer cases. Among 24 matched plasma samples, PTPRO was aberrantly methylated in 11 (45.8%) cases. Importantly and consistent with our findings, no methylation was observed in normal control plasma samples from 10 healthy individuals. These data help confirm that *PTPRO* methylation in plasma samples may provide a robust, specific, non-invasive means for early detection of breast cancer. However, the frequency of *PTPRO* methylation detected in plasma was lower than in cancer tissues and less association of methylation were found in plasma with clnicopathological data. This might due to fewer tumors DNA releasing in the circulation, or poor quality of DNA when extracted from peripheral blood, whose impact factors include acquisition condition, storage time, human factor, etc. Our method of detecting PTPRO methylation from plasma may not be extremely robust. The more standard conditions and a larger series of breast cancer patients should be involved for more understanding the molecular mechanism and clinical behavior of these tumors, as well as provide targets for better diagnosis and therapy. For sure, a more robust method must be used if this is translated to clinical application.

In agreement with You *et al*. [20], we found a strong correlation between *PTPRO* methylation and tumor stage (Table 2), with 84% of stage III tumors found to be methylated. Similar to Huang *et al*. [21], *PTPRO* methylation correlated with higher histological grade. The current study is the first to report an association between *PTPRO* methylation and positive lymph node status and *HER2* amplification in breast cancer. We also observed more frequent *PTPRO* methylation in ER-negative and PR-negative patient groups, possibly due to the association between these features and poor prognosis. Interestingly, Ramaswamy *et al*. [10] found that positive PTPRO expression was associated with improved response to tamoxifen; these results are consistent with previous reports of protein tyrosine phosphatase gene (PTPG) [35,36]. Therefore, estrogen-mediated suppression of *PTPRO* and the methylation of this gene may play important roles in estrogen-induced tumorigenesis. While interesting, each of the above associations with *PTPRO* methylation requires confirmation in larger studies. Moreover, it remains to be established whether the characteristic aggressive phenotype is linked to methylation via silencing of gene expression or through other mechanisms.

The significant associations between *PTPRO* methylation and nodal involvement, poorly differentiated histology, stage III tumors, and *HER2* amplification suggest that PTPRO expression may be involved in breast tumor invasion. Given these aforementioned correlations, it is not surprising that *PTPRO* methylation served as a prognostic indicator of worse outcome. Although *PTPRO* methylation was weakly associated with ER- and PR- status, these factors had no prognostic value in the current tumor series (data not shown). Similar to Huang *et al*. [21], unmethylated *PTPRO* was significantly associated with favorable outcome in ER + and PR + subgroups, as well as in patients with *HER2* amplification. As reported, activation of ER results in multiple downstream effects [37]. Recent studies indicate that ERβ expression is decreased in human neoplastic breast tissue, suggesting that ERβ may be an inhibitor of tumorigenesis [38-40]. For clinically apparent tumors, the proposed tumor-associated factors may help protect against tumor progression. Thus, according to prior studies, inactive PTPRO might be a stimulating factor during tumorigenesis, explain the ineffection of endocrine therapy and more precise subpopulations could be stratified to decide whether the patients with ER-positive need a regimen containing tamoxifen.

In a univariate model including strong prognostic factors such as nodal status, histological grade, tumor size, stage, *HER2* amplification, and *TP53* mutation status, *PTPRO* methylation of overall tumors, ER+, PR + and HER2+ group was found to be predictive of poorer outcome for breast cancer. Multivariate analysis identified methylated-*PTPRO* as an independent predictor for overall survival (*P =* 0.023), expecially in HER2+ subpopulation (*P =* 0.006). In contrast to our findings, Huang *et al*. reported that PTPRO methylation only correlated with higher histological grade but not with any other clinical parameters assessed [21]. This could be due to differences in sample size or to differences in sample processing. For example, while we used fresh tumor tissue, Huang et al. made use of formalin-fixed paraffin-embedded samples. Despite this, the trend is still in the same direction. That is, PTPRO methylation and low expression are associated with worse prognostic features, especially for HER2-positive patients. Further supporting our claim is work from another group showing that the receptor tyrosine kinase ErbB2/HER2 is a direct substrate of PTPRO, and low levels of PTPRO expression correlated with reduced survival of HER2-positive breast cancer patients [11]. This may also help explain why plasma PTPRO methylation was only significantly associated with HER2 amplification. The data we present here, in conjunction with earlier work, establish PTPRO as a likely tumor suppressor in breast cancer. Moreover, PTPRO methylation status might predict response to anti-HER-targeted therapies in HER2-positive patients, even provide extensive survival benefits or improve the efficiency of targeted drugs due to active PTPRO. To further study, patients who receive targeted therapy are required.

## Conclusion

In summary, our results confirm that *PTPRO* methylation is detected at a high frequency in breast cancer, occurring at a higher rate than either *TP53* mutation or *HER2* amplification. Positive associations with nodal involvement, poorly differentiated histology, and *HER2* amplification indicate that *PTPRO* methylation may contribute to an aggressive breast tumor phenotype. This was particularly evident for ER+, PR+, and *HER2*-amplified breast cancer subgroups, which all showed that *PTPRO* methylation in tumor tissues was a strong prognostic factor. Methylated-*PTPRO* could serve as an independent predictor for overall survival, expecially in HER2-positive breast cancer patients. Changes in protein tyrosine phosphatase activity likely play an important role in breast carcinogenesis and may provide a useful target for the development of novel therapies.

## Competing interests

The authors declare that they have no competing interests.

## Authors’ contributions

SL and RL developed the study and drafted the manuscript. YC and LR participated in sample collection and data analysis. LX and HW carried out the molecular genetic studies and participated in sequence alignment. *RL participated in the design of the study and its coordination and helped draft the manuscript. All authors read and approved the manuscript.

## References

[B1] Simpson PT Reis-Filho JS Gale T Lakhani SR Molecular evolution of breast cancer J Pathol 2005 205 248 254 10.1002/path.1691 15641021

[B2] Curigliano G Spitaleri G Dettori M Locatelli M Scarano E Goldhirsch A Vaccine immunotherapy in breast cancer treatment:Promising, but still early Expert Rev Anticancer Ther 2007 7 1225 1241 10.1586/14737140.7.9.1225 17892423

[B3] Emens LA Reilly RT Jaffee EM Augmenting the potency of breast cancer vaccines: Combined modality immunotherapy Breast Dis 2004 20 13 24 15687703 10.3233/bd-2004-20103

[B4] Dworkin AM Huang TH Toland AE Epigenetic alterations in the breast: Implications for breast cancer detection, prognosis and treatment Semin Cancer Biol 2009 19 165 171 10.1016/j.semcancer.2009.02.007 19429480 PMC2734184

[B5] Hesson LB Cooper WN Latif F The role of RASSF1A methylation in cancer Dis Markers 2007 23 73 87 10.1155/2007/291538 17325427 PMC3850810

[B6] Motiwala T Ghoshal K Das A Majumder S Weichenhan D Wu YZ Holman K James SJ Jacob ST Plass C Suppression of the protein tyrosine phosphatase receptor type O gene (PTPRO) by methylation in hepatocellular carcinomas Oncogene 2003 22 6319 6331 10.1038/sj.onc.1206750 14508512 PMC3020652

[B7] Motiwala T Majumder S Kutay H Smith DS Neuberg DS Lucas DM Byrd JC Grever M Jacob ST Methylation and silencing of protein tyrosine phosphatase receptor type O in chronic lymphocytic leukemia Clin Cancer Res 2007 13 3174 3181 10.1158/1078-0432.CCR-06-1720 17545520 PMC3074612

[B8] Motiwala T Kutay H Ghoshal K Bai S Seimiya H Tsuruo T Suster S Morrison C Jacob ST Protein tyrosine phosphatase receptor-type O (PTPRO) exhibits characteristics of a candidate tumor suppressor in human lung cancer Proc Natl Acad Sci U S A 2004 101 13844 13849 10.1073/pnas.0405451101 15356345 PMC518843

[B9] Hogan LE Meyer JA Yang J Wang J Wong N Yang W Condos G Hunger SP Raetz E Saffery R Relling MV Bhojwani D Morrison DJ Carroll WL Integrated genomic analysis of relapsed childhood acute lymphoblastic leukemia reveals therapeutic strategies Blood 2011 118 5218 5226 10.1182/blood-2011-04-345595 21921043 PMC3217405

[B10] Ramaswamy B Majumder S Roy S Wang J Wong N Yang W Condos G Hunger SP Raetz E Saffery R Relling MV Bhojwani D Morrison DJ Carroll WL Estrogen-mediated suppression of the gene encoding protein tyrosine phosphatase PTPRO in human breast cancer: mechanism and role in tamoxifen sensitivity Mol Endocrinol 2009 23 176 187 10.1210/me.2008-0211 19095770 PMC2646619

[B11] Yu M Lin G Arshadi N Kalatskaya I Xue B Haider S Nguyen F Boutros PC Elson A Muthuswamy LB Tonks NK Muthuswamy SK Expression profiling during mammary epithelial cell three-dimensional morphogenesis identifies PTPRO as a novel regulator of morphogenesis and ErbB2-mediated transformation Mol Cell Biol 2012 32 3913 3924 10.1128/MCB.00068-12 22851698 PMC3457532

[B12] Hunter T Signaling–2000 and beyond Cell 2000 100 113 127 10.1016/S0092-8674(00)81688-8 10647936

[B13] Fischer EH Cell signaling by protein tyrosine phosphorylation Adv Enzyme Regul 1999 39 359 369 10.1016/S0065-2571(98)00014-4 10470384

[B14] Laczmanska I Sasiadek MM Tyrosine phosphatases as a superfamily of tumor suppressors in colorectal cancer Acta Biochim Pol 2011 V58N4 467 470 22146137

[B15] Gupta S Radha V Sudhakar C Swarup G A nuclear protein tyrosine phosphatase activates p53 and induces caspase-1-dependent apoptosis FEBS Lett 2002 532 61 66 10.1016/S0014-5793(02)03628-1 12459463

[B16] Salmeen A Andersen JN Myers MP Tonks NK Barford D Molecular basis for the dephosphorylation of the activation segment of the insulin receptor by protein tyrosine phosphatase 1B Mol Cell 2000 6 1401 1412 10.1016/S1097-2765(00)00137-4 11163213

[B17] Alonso A Sasin J Bottini N Friedberg I Friedberg I Osterman A Godzik A Hunter T Dixon J Mustelin T Protein tyrosine phosphatases in the human genome Cell 2004 117 699 711 10.1016/j.cell.2004.05.018 15186772

[B18] Andersen JN Mortensen OH Peters GH Drake PG Iversen LF Olsen OH Jansen PG Andersen HS Tonks NK Møller NP Structural and evolutionary relationships among protein tyrosine phosphatase domains Mol Cell Biol 2001 21 7117 7136 10.1128/MCB.21.21.7117-7136.2001 11585896 PMC99888

[B19] Jacob ST Motiwala T Epigenetic regulation of protein tyrosine phosphatases: potential molecular targets for cancer therapy Cancer Gene Ther 2005 12 665 672 10.1038/sj.cgt.7700828 15803146 PMC3028596

[B20] You YJ Chen YP Zheng XX Meltzer SJ Zhang H Aberrant methylation of the PTPRO gene in peripheral blood as a potential biomarker in esophageal squamous cell carcinoma patients Cancer Lett 2012 315 138 144 10.1016/j.canlet.2011.08.032 22099875 PMC3248961

[B21] Huang YT LIFF Ke C Li Z Li ZT Zou XF Zheng XX Chen YP Zhang H PTPRO promoter methylation is predictive of poorer outcome for HER2-positive breast cancer: indication for personalized therapy J Transl Med 2013 11 245 10.1186/1479-5876-11-245 24090193 PMC3852714

[B22] Sobin LH Wittekind C International Union Against Cancer (UICC), 5th ed., TNM classification of malignant tumors 1997 Baltimore (MD): Wiley-Liss 54 58

[B23] Abd El-Rehim DM Bal G Pinder SE Rakha E Paish C Robertson JF Macmillan D Blamey RW Ellis IO High-throughput protein expression analysis using tissue microarray technology of a large well-characterised series identifies biologically distinct classes of breast cancer confirming recent cDNAexpression analyses Int J Cancer 2005 116 340 350 10.1002/ijc.21004 15818618

[B24] Li SY Rong MN Iacopetta B DNA hypermethylation in breast cancer and its association with clinicopathological features Cancer Lett 2006 237 272 280 10.1016/j.canlet.2005.06.011 16029926

[B25] Herman JG Graff JR Myohanen S Nelkin BD Baylin SB Methylation-specific PCR: a novel PCR assay for methylation status of CpG islands Proc Natl Acad Sci U S A 1996 93 9821 9826 10.1073/pnas.93.18.9821 8790415 PMC38513

[B26] Paulin R Grigg G Davey MW Piper AA Urea improves efficiency of bisulphate-mediated sequencing of 50-methylcytosine in genomic DNA Nucleic Acids Res 1998 26 5009 5010 10.1093/nar/26.21.5009 9776768 PMC147923

[B27] Motiwala T Jacob ST Role of protein tyrosine phosphatases in cancer Prog Nucleic Acid Res Mol Biol 2006 81 297 329 16891175 10.1016/S0079-6603(06)81008-1 PMC3077959

[B28] Tonks NK Protein tyrosine phosphatases: from genes, to function, to disease Nat Rev Mol Cell Biol 2006 7 833 846 10.1038/nrm2039 17057753

[B29] Ostman A Hellberg Cand Bohmer FD Protein-tyrosine phosphatases and cancer Nat Rev Cancer 2006 6 307 320 10.1038/nrc1837 16557282

[B30] Hsu SH Motiwala T Roy S Claus R Mustafa M Plass C Freitas MA Ghoshal K Jacob ST Methylation of the PTPRO gene in human hepatocellular carcinoma and identification of VCP as its substrate J Cell Biochem 2013 114 1810 1818 10.1002/jcb.24525 23533167 PMC4199230

[B31] Motiwala T Majumder S Ghoshal K Kutay H Datta J Roy S Lucas DM Jacob ST PTPROt inactivates the oncogenic fusion protein BCR/ABL and suppresses transformation of K562 cells J Biol Chem 2009 284 455 464 10.1074/jbc.M802840200 18997174 PMC2610515

[B32] Jin Z Cheng Y Olaru A Kan T Yang J Paun B Ito T Hamilton JP David S Agarwal R Selaru FM Sato F Abraham JM Beer DG Mori Y Shimada Y Meltzer SJ Promoter hypermethylation of CDH13 is a common, early event in human esophageal adenocarcinogenesis and correlates with clinical risk factors Int J Cancer 2008 123 2331 2336 10.1002/ijc.23804 18729198

[B33] Jin Z Hamilton JP Yang J Mori Y Olaru A Sato F Ito T Kan T Cheng Y Paun B David S Beer DG Agarwal R Abraham JM Meltzer SJ Hypermethylation of the AKAP12 promoter is a biomarker of Barrett’sassociated esophageal neoplastic progression Cancer Epidemiol Biomarkers Prev 2008 17 111 117 10.1158/1055-9965.EPI-07-0407 18199717

[B34] Jin Z Olaru A Yang J Sato F Cheng Y Kan T Mori Y Mantzur C Paun B Hamilton JP Ito T Wang S David S Agarwal R Beer DG Abraham JM Meltzer SJ Hypermethylation of tachykinin-1 is a potential biomarker in human esophageal cancer Clin Cancer Res 2007 13 6293 6300 10.1158/1078-0432.CCR-07-0818 17975140

[B35] Liu S Sugimoto Y Sorio C Tecchio C Lin YC Function analysis of estrogenically regulated protein tyrosine phosphatase (PTPs) in human breast cancer cell line MCF-7 Oncogene 2004 23 1256 1262 10.1038/sj.onc.1207235 14676845

[B36] Zheng J Kulp SK Zhang Y Sugimoto Y Dayton MA Govindan MV Brueggemeier RW Lin YC 17-Estradiol-regulated expression of protein tyrosine phosphatase gene in cultured human normal breast and breast cancer cells Anticancer Res 2000 20 11 19 10769629

[B37] Henderson BE Feigelson HS Hormonal carcinogenesis Carcinogenesis 2000 21 427 433 10.1093/carcin/21.3.427 10688862

[B38] Dotzlaw H Leygue E Watson PH Murphy LC Estrogen receptor messenger RNA expression in human breast tumor biopsies: relationship to steroid receptor status and regulation by progestins Cancer Res 1999 59 529 532 9973194

[B39] Iwao K Miyoshi Y Egawa C Ikeda N Noguchi S Quantitative analysis of estrogen receptor-β mRNA and its variants in human breast cancers Int J Cancer 2000 88 733 736 10.1002/1097-0215(20001201)88:5<733::AID-IJC8>3.0.CO;2-M 11072241

[B40] Roger P Sahla ME Makela S Gustafsson JA Baldet P Rochefort H Decreased expression of estrogen receptor-β protein in proliferative preinvasive mammary tumors Cancer Res 2001 61 2537 2541 11289127

